# Smoking-diseases correlation database: comprehensive analysis of the correlation between smoking and 422 diseases based on NHANES 2013–2018

**DOI:** 10.3389/fpubh.2024.1325856

**Published:** 2024-06-07

**Authors:** Xi Chen, Tengkun Wang, Yushan Tian, Yinchao Ma, Yuan Liu, Huan Chen, Hongwei Hou, Qingyuan Hu, Ming Chu

**Affiliations:** ^1^NHC Key Laboratory of Medical Immunology (Peking University), Department of Immunology, School of Basic Medical Sciences, Peking University, Beijing, China; ^2^Department of Adult Joint Reconstructive Surgery, Beijing Jishuitan Hospital, Capital Medical University, Beijing, China; ^3^School of Public Health, Peking University, Beijing, China; ^4^Beijing Life Science Academy, Beijing, China

**Keywords:** smoking, smoking-diseases correlation database, smoking-associated diseases, smoking positively associated diseases, smoking negatively associated diseases

## Abstract

**Background:**

Smoking is a risk factor for a wide range of diseases. Previous research has confirmed over 30 Smoking-Associated Diseases in diverse systems. There is limited research exploring the correlation among multiple diseases, with an absence of comprehensive investigations. Few studies concentrate on diseases exhibiting a negative correlation with smoking, wherein smokers demonstrate a lower prevalence.

**Objective:**

This study aimed to detect the correlation between smoking and other diseases using data from National Health and Nutrition Examination Surveys (NHANES) and construct a Smoking-Diseases Correlation Database (SDCD). The second aim is to obtain an extensive screening test for diseases that may be linked to smoking.

**Methods:**

39,126 subjects’ data from the NHANES 2013–2018 dataset were extracted. The baseline information, difference in blood routine and blood chemistry indicators between smokers and non-smokers, and diseases’ correlation with smoking in four different models were analyzed by R. The data and statistics were aggregated into an online SDCD.

**Results:**

Our study reported 46 Smoking-Associated Diseases (SAD), including 29 Smoking Positively Associated Diseases (SPAD) and 17 Smoking Negatively Associated Diseases (SNAD). The SDCD of 422 diseases was constructed and can be accessed at https://chatgptmodel.shinyapps.io/sdcd/.

**Conclusion:**

Our findings revealed 46 SADs including 29 SPADs and 17 SNADs. We aggregated the statistics and developed online SDCD, advancing our understanding of the correlation between smoking and diseases.

## 1 Introduction

Smoking is the main cause of preventable early death around the world. Approximately one person dies every 6 s due to smoking, accounting for one in five deaths in the age group between 35 and 69 (1, 2). According to the WHO, the total number of tobacco users is 1.30 billion in 2020, and the prevalence rate of tobacco use is 22.3% of the global population. Over 8 million people died from tobacco-related diseases in 2019 (3). In the 1950s, Ernst Wynder et al. put forward a hypothesis that lung cancer is associated with smoking. As the evidence accumulated, in 1964, a report from the United States Department of Health confirmed the causal relationship between smoking and lung cancer (4). From then on, a quantity of evidence was regularly updated and more Smoking-Associated Diseases (SAD) were found. Studies have established that smoking can affect different organs and systems such as cardiovascular (5), respiratory (6), brain (7), reproductive (8), immune (9) systems, etc. The number of SADs increases gradually and has reached at least 30, but there are still SADs that are not being detected or lack enough evidence (10).

Tremendous research focuses on Smoking Positively Associated Diseases (SPAD). However, studies have also found that some diseases were Smoking Negatively Associated Diseases (SNAD). For example, smokers have a lower prevalence of Parkinson’s disease than nonsmokers, which is consistently shown by previous epidemiology research. Because combustible tobacco smoke has been seen as a cause of adverse health outcomes, this inverse correlation was counterintuitive and fascinating (11). Furthermore, studies suggest that non-smokers have a relatively higher risk of ulcerative colitis and osteoarthritis than smokers (12). If we were to document favorable effects of smoking on different diseases, this might provide an opportunity to identify constituents of combustible tobacco smoke that have favorable effects on disease and to test these as potential treatments (12).

The majority of present studies are dispersed and specialize in a certain disease, and lack comprehensive research including multiple diseases in one study. The comprehensive study is important for the understanding of tobacco’s effect on health. Therefore, we evaluated the correlation between smoking and 422 different diseases using data of 39,126 people from the National Health and Nutrition Examination Survey dataset of the United States (NHANES) and paid attention to both SPAD and SNAD. By extracting and analyzing a giant collection of data, knowledge of this database could provide a new landscape on smoking and disease, laying a foundation for further research on SAD.

## 2 Methods

### 2.1 Study population

For this investigation, we utilized the data extracted from the National Health and Nutrition Examination Survey dataset of the United States (NHANES) spanning from 2013 to 2018. All the data we used could be accessed at https://wwwn.cdc.gov/nchs/nhanes/Default.aspx. 12,226 individuals were excluded from the study due to missing or incomplete data on body mass index (BMI), education level, smoking records, and uncertain or incomplete information. Additionally, 24 individuals who lacked ICD-10 diagnosis information were also ruled out in this study. A total of 39,126 subjects remained for the final analysis. The study of NHANES 2013–2018 was performed according to the guidelines approved by the Research Ethics Review Board of the National Center for Health Statistics (Protocol #2011-17, #2018-01). Moreover, written informed consent was duly obtained from all participants involved in the NHANES project.

### 2.2 Study variables

Variables data from NHANES is collected through a combination of interviews, physical examinations, and laboratory tests. Participants are interviewed to gather information on their medical history, lifestyle factors, and dietary habits. Additionally, a comprehensive physical examination is conducted, which includes measuring vital signs and collecting blood samples. The blood samples are then analyzed in a laboratory, where tests are performed to measure the various parameters of interest. These data contribute to the rich dataset available in the NHANES database, which allows researchers and public health professionals to study and monitor the health and nutritional status of the United States population.

National Health and Nutrition Examination Surveys employs a weighted sampling approach to ensure its findings are representative of the United States population. Participants are assigned base weights reflecting their probability of selection. Nonresponse adjustments account for those who choose not to participate, and post-stratification aligns the sample with known population demographics.

The population variables involved in this study include age, BMI, sex, race, and education level. Ethnicity and race are determined by a series of questions in interviews. Hispanic or Latino refers to anyone who says they were born in or had ancestors from Spain or one of the western hemisphere territories or countries where Spanish is the primary language. The blood routine and blood chemistry indicators involved in this study include white blood cell count (WBC), neutrophils (NEU), lymphocytes (LYM), monocytes (MO), platelets (PLT), neutrophil-to-lymphocyte ratio (NLR), monocyte-to-lymphocyte ratio (MLR), systemic immune inflammation index (SII), systemic immune response index (SIRI), alkaline phosphatase (ALP), total bilirubin (TBIL), albumin (ALB), gamma-glutamyl transferase (GGT), iron (Fe), and alpha-klotho (a-klotho).

The dependent variables of this research were 422 diseases with ICD-10 coding reported by each individual’s response to the questionnaire using the following question: “Has a doctor ever diagnosed you with….” 422 diseases involved multiple organs and systems cover the smoking’s influence on health as comprehensive as possible.

In this study, the smoking population is delineated as individuals who have consumed a minimum of 100 cigarettes throughout their lifetime. This definition is consistent with the report methods of other diseases in the questionnaire in order to get a better correlation analysis.

### 2.3 Statistical analyses

We have performed all statistical analysis with the R software (Version 4.2.2) according to the CDC guidelines.^1^ Sample weights were taken into account in all of the estimates to produce representative data of the civilian noninstitutionalized United States population. The correlation between smoking and each disease was assessed through four multivariable logistic regression models: one unadjusted and three adjusted models. Model 1 did not include any covariate adjustments. In Model 2, adjustments were made for age, gender, and race. Model 3 accounted for age, gender, race, education level, and poverty-to-income ratio. Finally, Model 4 incorporated adjustments for age, gender, race, education level, poverty-to-income ratio, smoking status, body mass index, and bone mineral density.

We constructed a Smoking-Diseases Correlation Database (SDCD), an online repository of smoking-disease correlations, using the R software package Shiny and the statistics. The database can be accessed at the following web address: https://chatgptmodel.shinyapps.io/sdcd/. There are the characteristics of the participants, the association between smoking and selected diseases, and subgroup analysis data on the website. Subgroup analysis refers to an analysis of OR values of diseases among smokers relative to non-smokers in different gender and ethnic groups separately.

## 3 Results

### 3.1 Baseline information of study participants

A total of 39,126 participants (mean age 53.90 ± 0.35, 46% males) were included in this study. 48.18% of the participants have a history of smoking. The weighted distribution of the characteristics according to smoking is shown in Table 1. The average age and BMI in these two groups did not have a significant difference. Regarding sex and race, Males and non-Hispanic white people were more likely to have a higher rate of smoking. There are more smokers than non-smokers in the College or above and the High School or equivalent education level participants groups and fewer smokers than non-smokers in the College graduate or above and the Less than high school groups.

**Table 1:** Baseline information according to smoking or not smoking of NHANES data from 2013 to 2018.

Variable	Smoker	Non-smoker	*p* value
Age	54.63 ± 0.42	52.25 ± 0.42	0.12
BMI	30.86 ± 0.24	30.38 ± 0.22	0.11
Sex			< 0.0001
Female	8,228 (47.19)	12,899 (62.71)	
Male	10,623 (52.81)	7,376 (37.29)	
Race			< 0.0001
Mexican American	1964 (5.40)	3,062 (8.32)	
Non-Hispanic Black People	4,022 (9.63)	4,204 (11.28)	
Non-Hispanic White People	9,367 (73.15)	7,497 (65.42)	
Others	3,498 (11.81)	5,512 (14.97)	
Education			< 0.0001
College graduate or above	2,887 (20.67)	5,378 (34.45)	
College or above	6,132 (34.52)	5,796 (30.39)	
High School or equivalent	7,971 (39.74)	5,912 (26.99)	
Less than high school	1861 (5.06)	3,189 (8.18)	
Total	18,851	20,275	

Figures in parentheses correspond to the weighted column percentage.

### 3.2 Correlations between smoking and blood routine and blood biochemistry variables

We conducted a study examining the disparities in blood routine and blood biochemistry variables between smokers and non-smokers (Table 2). To assess the difference between smokers and non-smokers, a *t*-test was computed. Compared to the non-smoking population, the majority of immunological inflammatory markers were significantly elevated in the smoking population, including white blood cell count (WBC), neutrophil count (NEU), lymphocyte count (LYM), monocyte count (MO), neutrophil-to-lymphocyte ratio (NLR), monocyte-to-lymphocyte ratio (MLR), systemic immune-inflammation index (SII), and systemic immune response index (SIRI). Additionally, there were notable increases in certain oxidative stress indicators such as Alkaline Phosphatase (ALP), albumin (ALB), Gamma-Glutamyl Transferase (GGT), and decreases of α-Klotho and total bilirubin (TBIL), in the smoking population.

**Table 2:** The correlation between blood routine and blood biochemistry indicators and smoking.

Indicators	Mean ± SE		*p* value
	Smoker	Non-smoker	
WBC	7.91 ± 0.08	7.26 ± 0.06	< 0.0001
NEU	4.76 ± 0.05	4.30 ± 0.04	< 0.0001
LYM	2.25 ± 0.05	2.13 ± 0.02	0.01
MO	0.63 ± 0.00	0.58 ± 0.00	< 0.0001
PLT	236.10 ± 2.16	239.63 ± 1.37	0.08
NLR	2.43 ± 0.03	2.25 ± 0.03	< 0.0001
MLR	0.32 ± 0.00	0.30 ± 0.00	< 0.0001
SII	570.82 ± 6.95	536.37 ± 6.42	< 0.001
SIRI	0.01 ± 0.00	0.01 ± 0.00	< 0.0001
ALP	74.63 ± 1.14	71.44 ± 0.52	0.01
TBIL	0.52 ± 0.01	0.55 ± 0.01	< 0.0001
ALB	4.16 ± 0.02	4.19 ± 0.01	0.05
GGT	34.50 ± 0.84	27.21 ± 0.70	< 0.0001
Fe	138.35 ± 4.94	128.86 ± 3.38	0.07
a-klotho	795.82 ± 10.16	830.93 ± 8.71	0.01

Among non-smokers, for example, the WBC was 7.26, while this rose 9% to 7.91 in former smokers. NEU indicator rises 11% in smokers. MO rises 9%. Smokers’ GGT indicator (34.50) is 27% larger than non-smokers (27.21) and it has the biggest increase percentage in all risen blood indicators. Interestingly, TBIL and a-klotho, respectively, decreased by 5 and 4%, and they are both the only decreased indicators with statistical significance.

### 3.3 Positive correlation between smoking and diseases

Within this investigation, we delved into the correlation between smoking and various diseases. We examined a comprehensive collection of 422 prevalent illnesses categorized by the ICD-10 classification system and calculated the disparities in disease incidence between smokers and non-smokers. Ultimately, we identified 29 diseases that exhibited a significant positive correlation with smoking (Table 3). SPADs listed in Table 3 primarily focus on COPD, mental health disorders, pain, and gastrointestinal diseases. It also includes muscle spasm.

**Table 3:** SPADs’ names, ICD-10 numbers, and OR values among smokers relative to non-smokers in four models after adjusting for variables of interest.

ICD-10	Diseases name	OR (95%CI)			
		Model 1^a^	Model 2^b^	Model 3 ^c^	Model 4^d^
J44.9	Chronic obstructive pulmonary disease, unspecified	7.77 (4.82, 12.52)	7.33 (4.57, 11.77)	6.19 (3.71, 10.32)	6.94 (4.08, 11.82)
R45.0	Nervousness	5.27 (3.20, 8.68)	5.84 (3.56, 9.59)	3.46 (1.40, 8.55)	2.90 (0.75, 11.20)
F20	Schizophrenia	4.65 (2.29, 9.44)	5.28 (2.55, 10.94)	3.70 (1.68, 8.14)	4.73 (1.97, 11.34)
E78.1	Pure hyperglyceridemia	3.83 (1.16, 12.63)	2.48 (0.76, 8.03)	2.93 (0.88, 9.82)	2.80 (0.82, 9.53)
F42	Obsessive-compulsive disorder	3.69 (1.04, 13.16)	3.85 (0.99, 14.86)	4.82 (0.94, 24.68)	4.84 (0.93, 25.22)
M54.2	Cervicalgia	3.48 (1.12, 10.83)	3.77 (1.11, 12.82)	3.51 (1.00, 12.32)	3.49 (0.93, 13.12)
K25	Gastric ulcer	3.39 (1.48, 7.80)	3.66 (1.67, 8.02)	4.25 (1.79, 10.10)	4.08 (1.74, 9.58)
I20.9	Angina pectoris, unspecified	3.37 (1.64, 6.92)	2.77 (1.36, 5.64)	2.28 (1.05, 4.93)	2.44 (1.07, 5.56)
R19.7	Diarrhea, unspecified	3.34 (1.29, 8.66)	3.28 (1.28, 8.36)	4.17 (1.73, 10.06)	3.67 (1.42, 9.48)
M25.51	Pain in shoulder	3.19 (1.12, 9.12)	3.45 (1.24, 9.59)	3.33 (1.13, 9.78)	3.52 (1.06, 11.62)
F29	Unspecified psychosis not due to a substance or known physiological condition	3.17 (1.20, 8.37)	3.53 (1.16, 10.76)	3.54 (0.87, 14.45)	3.49 (0.85, 14.27)
F43.1	Post-traumatic stress disorder (PTSD)	3.02 (1.37, 6.64)	3.06 (1.34, 6.95)	2.74 (1.13, 6.63)	2.73 (1.13, 6.61)
K27	Peptic ulcer, site unspecified	2.93 (1.24, 6.96)	3.22 (1.28, 8.09)	3.12 (1.12, 8.72)	4.00 (1.31, 12.17)
M54.9	Dorsalgia, unspecified	2.28 (1.53, 3.40)	2.25 (1.54, 3.29)	1.88 (1.25, 2.84)	1.81 (1.16, 2.81)
R06.9	Unspecified abnormalities of breathing	2.24 (1.03, 4.84)	2.00 (0.88, 4.51)	1.73 (0.69, 4.34)	1.77 (0.68, 4.64)
R12	Heartburn	2.08 (1.20, 3.62)	1.98 (1.12, 3.52)	1.91 (1.04, 3.50)	1.78 (0.93, 3.39)
M62.83	Muscle spasm	2.05 (1.34, 3.14)	2.14 (1.37, 3.34)	1.86 (1.15, 2.99)	2.02 (1.26, 3.25)
K08.8	Other specified disorders of teeth and supporting structures	1.97 (0.91, 4.28)	2.38 (1.11, 5.08)	2.07 (0.94, 4.54)	2.03 (0.92, 4.47)
M79.2	Neuralgia and neuritis, unspecified	1.70 (1.27, 2.27)	1.68 (1.24, 2.26)	1.54 (1.08, 2.19)	1.65 (1.16, 2.35)
N40	Enlarged prostate	1.58 (1.10, 2.28)	0.85 (0.58, 1.25)	0.82 (0.56, 1.20)	0.77 (0.52, 1.16)
T14.9	Unspecified injury	1.48 (1.07, 2.05)	1.63 (1.16, 2.29)	1.48 (1.06, 2.08)	1.40 (0.97, 2.03)
I50.9	Heart failure, unspecified	1.45 (1.04, 2.01)	1.22 (0.86, 1.73)	1.16 (0.81, 1.66)	1.25 (0.86, 1.82)
F32.9	Major depressive disorder, single episode, unspecified	1.41 (1.22, 1.62)	1.52 (1.32, 1.75)	1.49 (1.29, 1.73)	1.51 (1.30, 1.74)
G47.9	Sleep disorder, unspecified	1.38 (1.04, 1.83)	1.38 (1.05, 1.81)	1.32 (0.99, 1.78)	1.38 (1.00, 1.92)
R73	Elevated blood glucose level	1.36 (1.04, 1.77)	1.15 (0.88, 1.51)	1.16 (0.88, 1.53)	1.12 (0.85, 1.49)
G47.0	Insomnia	1.35 (1.10, 1.66)	1.39 (1.14, 1.71)	1.27 (1.02, 1.58)	1.26 (1.00, 1.57)
F41.9	Anxiety disorder, unspecified	1.34 (1.15, 1.56)	1.45 (1.24, 1.68)	1.43 (1.21, 1.70)	1.45 (1.22, 1.71)
K21	Gastro-esophageal reflux disease	1.18 (1.01, 1.37)	1.12 (0.96, 1.30)	1.07 (0.89, 1.28)	1.08 (0.90, 1.29)
E78.0	Pure hypercholesterolemia	1.16 (1.06, 1.28)	0.99 (0.89, 1.09)	1.01 (0.91, 1.14)	0.98 (0.87, 1.11)

^a^Model 1 adjusted for no covariates.

^b^Model 2 adjusted for age, gender, and race.

^c^Model 3 adjusted for age, gender, race, education level, and poverty to income ratio.

^d^Model 4 adjusted for age, gender, race, education level, poverty to income ratio, body mass index, and bone mineral density.

### 3.4 Negative correlation between smoking and diseases

Notably, we also identified 17 diseases that displayed a significant negative correlation with smoking (Table 4). SNADs listed in Table 4 cover a broader range of diseases across various medical domains, including infectious diseases, endocrine disorders (diabetes), neurological conditions (Parkinson’s disease), eye disorders, respiratory conditions (pulmonary embolism, otitis media), musculoskeletal conditions (psoriasis, osteoporosis), and genitourinary disorders (kidney failure, menstrual irregularities).

**Table 4:** SNADs’ names, ICD-10 numbers, and OR values among smokers relative to non-smokers in four models after adjusting for variables of interest.

ICD-10	Diseases name	OR (95%CI)			
		Model 1 ^a^	Model 2 ^b^	Model 3 ^c^	Model 4 ^d^
J00	Acute nasopharyngitis [common cold]	0.11 (0.03, 0.43)	0.13 (0.03, 0.50)	0.13 (0.03, 0.61)	0.13 (0.03, 0.61)
A49.9	Bacterial infection	0.14 (0.02, 0.82)	0.13 (0.02, 0.94)	0.10 (0.01, 1.25)	0.11 (0.01, 1.36)
E63.9	Nutritional deficiency, unspecified	0.15 (0.02, 0.97)	0.10 (0.01, 0.79)	0.14 (0.02, 1.11)	0.14 (0.02, 1.12)
T86.9	Transplanted organ and tissue rejection	0.20 (0.08, 0.52)	0.18 (0.06, 0.50)	0.14 (0.04, 0.49)	0.14 (0.04, 0.50)
L70	Acne	0.22 (0.09, 0.53)	0.39 (0.18, 0.86)	0.55 (0.24, 1.27)	0.61 (0.27, 1.37)
Z79.3	Long term (current) use of hormonal contraceptives	0.25 (0.17, 0.36)	0.44 (0.31, 0.63)	0.62 (0.43, 0.90)	0.65 (0.45, 0.94)
I26	Pulmonary embolism	0.26 (0.08, 0.80)	0.23 (0.07, 0.74)	0.24 (0.08, 0.72)	0.25 (0.07, 0.85)
R41.3	Other amnesia	0.28 (0.03, 2.55)	0.27 (0.02, 4.27)	0.04 (0.00, 0.56)	0.04 (0.00, 0.57)
N92	Excessive, frequent and irregular menstruation	0.31 (0.16, 0.59)	0.63 (0.34, 1.20)	0.70 (0.37, 1.32)	0.68 (0.37, 1.25)
H66.9	Otitis media, unspecified	0.32 (0.11, 0.93)	0.37 (0.12, 1.12)	0.46 (0.17, 1.29)	0.46 (0.17, 1.29)
H57.9	Unspecified disorder of eye and adnexa	0.33 (0.14, 0.75)	0.30 (0.10, 0.86)	0.30 (0.10, 0.90)	0.28 (0.09, 0.89)
N19	Unspecified kidney failure	0.35 (0.15, 0.85)	0.37 (0.16, 0.87)	0.38 (0.14, 0.99)	0.39 (0.14, 1.06)
G43	Migraine	0.39 (0.19, 0.79)	0.48 (0.22, 1.03)	0.65 (0.29, 1.45)	0.61 (0.27, 1.38)
G20	Parkinson’s disease	0.61 (0.31, 1.18)	0.49 (0.22, 1.11)	0.40 (0.19, 0.84)	0.36 (0.13, 0.97)
J30.9	Allergic rhinitis, unspecified	0.62 (0.41, 0.93)	0.64 (0.42, 0.96)	0.75 (0.48, 1.19)	0.76 (0.48, 1.22)
M81	Osteoporosis without current pathological fracture	0.62 (0.41, 0.95)	0.81 (0.52, 1.28)	0.85 (0.55, 1.30)	0.80 (0.53, 1.21)
E11.2	Type 2 diabetes mellitus with kidney complications	0.64 (0.31, 1.28)	0.49 (0.25, 0.98)	0.44 (0.20, 0.95)	0.43 (0.20, 0.93)

^a^Model 1 adjusted for no covariates.

^b^Model 2 adjusted for age, gender, and race.

^c^Model 3 adjusted for age, gender, race, education level, and poverty to income ratio.

^d^Model 4 adjusted for age, gender, race, education level, poverty to income ratio, body mass index, and bone mineral density.

## 4 Discussion

Blood routine and blood biochemistry indicators had 12 differences between smokers and non-smokers, with the majority of indicators showing higher levels in smokers. Elevated indicators suggest an increased inflammatory load and oxidative stress in smokers. One protein α-klotho caught our attention. Data showed that the α-klotho level in smokers is 4% lower than in non-smokers. Klotho gene is one of the most important genes related to the aging process and controls many metabolic pathways. Klotho protein has anti-aging, anti-inflammatory, anti-oxidant, and cardioprotective effects. A study suggested that oxidative stress from smoking can disrupt the anti-oxidant defense and decrease α-klotho protein synthesis (13).

Notably, our study identified 29 diseases possibly with a positive correlation with smoking. The most prominent one of these 29 SPADs is COPD, while many SPADs can be categorized into groups by their features, the mechanism of which requires further research, such as mental disorders, pain, and gastrointestinal disorders.

Chronic obstructive pulmonary disease has the highest OR value, respectively 7.77, 7.33, 6.19, and 6.94 among both four models and 29 SPADs. COPD is commonly associated with smoking; in addition to that, second-hand smoke, occupational exposures, air pollutants, and a history of previous lung infections are risk factors (14). One possible mechanism is that cigarette smoke can reprogram the epithelium of trachea and cause mitochondrial dysfunction (14, 15). A perspective suggested that COPD is a polygenetic disease, with complex environment risk factors accumulating through lifetime and interacting with susceptible genes (16).

Many mental disorders are SPADs. The rate of smoking among schizophrenia patients is 4.65 times higher compared to the general population, and other research indicates rates ranging from 2 to 5 times higher (17, 18). Several hypotheses have been proposed to explain this correlation, including (a) the causal relationship hypothesis, (b) the self-medication hypothesis, and (c) the shared genetic background hypothesis (17–20). In addition to schizophrenia, patients with nervousness, anxiety, and depression have respective OR values of 5.27, 1.34, and 1.41 in model 1.

Pain constitutes another category of SPADs that we have reported. Individuals experiencing chronic pain are approximately twice as likely to be current smokers compared to those without chronic pain (21). Firstly, nicotine exhibits an acute analgesic effect, intensifying pain in nicotine-deprived smokers and thereby making smoking cessation efforts more difficult (22). Secondly, smokers may be driven to use tobacco as a response to pain through intricate psychological mechanisms. An interactive reciprocal model of pain and smoking suggests that pain and smoking form a positive feedback loop, resulting in exacerbated pain and increased smoking (23).

More interestingly, we identified 18 different SNADs in this study, in which Parkinson’s disease (PD), allergic rhinitis, and long-term use of hormonal contraceptives (OCs) aroused our interest.

The OR value for Parkinson’s disease is 0.36 in model 4. Numerous epidemiological studies have consistently reported the same negative result. This correlation exhibits dose-dependent and time-dependent characteristics: RRs of PD were 0.8, 0.6, 0.5, and 0.4, for 1–9, 10–24, 25–44, and 45+ pack-years, respectively, relative to never-smokers (11). Notably, there have been compelling evidence for the relationship between smoking and the declining prevalence of PD. Arguments against this correlation, such as confounding by unknown factors, shared genes, and personality characteristics of PD patients, remain unlikely (24). The neuroprotective role shown by smoking might be related to the biological effect of nicotine, which has demonstrated neuroprotective effects in animal models of PD (25).

Allergic rhinitis showed a significant negative correlation in models 1 and 2 but did not show significance in models 3 and 4. The OR values were comparable between model 1 (0.62) and 2 (0.64), as well as between 3 (0.75) and 4 (0.76). A two-sample Mendelian randomization study also revealed that smoking can decrease the risk of allergic rhinitis and increase the risk of vasomotor rhinitis. One hypothesis suggested that individuals with allergic rhinitis may have a preference for smoking. Another hypothesis suggested that smoking might inhibit the immune system, thereby reducing the risk of allergic rhinitis (26).

Long-term use of hormonal contraceptives has a low prevalence (OR = 0.44) in smokers after gender was adjusted in model 2. Currently, there is limited understanding of the correlation between OCs’ use and smoking, with conflicting data reported in various studies. The most common OCs, which contain estrogen and progesterone components, can alter body progesterone and estradiol levels. Former studies have explored the potential impact of OCs on nicotine metabolism, cardiovascular reactivity, mood, and withdrawal (27). Some studies have investigated the clinical trial of exogenous progesterone’s effect on smoking cessation. One study discovered that exogenous progesterone increased the odds of quitting in females nearly 3-fold and averaged 6 more days before relapse (28). The proposition of exogenous progesterone, either as an adjunctive or stand-alone therapy for smoking cessation has been put forward (29). Further epidemiological studies are required to better understand the correlation between OCs and tobacco use.

## 5 Conclusion

Based on NHANES 2013–2018, we made a comprehensive analysis of the correlation between smoking and 422 diseases and found 46 SADs including 29 SPADs. Interestingly, we revealed 17 SNADs such as Parkinson’s disease, allergic rhinitis, and long-term use of hormonal contraceptives. It is noted that we put forward some previously not well-known Smoking-Associated Diseases, which require further research. Therefore, we constructed the online SDCD and summed up the correlation data between 422 diseases and smoking, making it easy to find. Our SDCD proves the importance of a large database in smoking research. These results facilitated our understanding of the correlation between smoking and diseases, providing the basis for studying the correlation between smoking and diseases.

## Data availability statement

The datasets presented in this study can be found in online repositories. The names of the repository/repositories and accession number(s) can be found at: https://chatgptmodel.shinyapps.io/sdcd/.

## Ethics statement

The studies involving humans were approved by the National Center for Health Statistics. The studies were conducted in accordance with the local legislation and institutional requirements. Written informed consent for participation in this study was provided by the participants’ legal guardians/next of kin.

## Author contributions

XC: Formal analysis, Methodology, Software, Writing – original draft. TW: Formal analysis, Visualization, Writing – original draft. YT: Data curation, Resources, Writing – review & editing. YM: Writing – review & editing. YL: Writing – review & editing. HC: Funding acquisition, Writing – review & editing. HH: Funding acquisition, Writing – review & editing. QH: Writing – review & editing, Funding acquisition. MC: Writing – review & editing, Conceptualization, Project administration.

## Footnotes

^1^ https://wwwn.cdc.gov/nchs/nhanes/tutorials
